# General practitioners’ perceptions of best practice care at the end of life: a qualitative study

**DOI:** 10.3399/bjgpopen19X101660

**Published:** 2019-09-04

**Authors:** Anne Herrmann, Mariko Carey, Alison Zucca, Lucy Boyd, Bernadette Roberts

**Affiliations:** 1 Conjoint Lecturer, Health Behaviour Research Collaborative, School of Medicine and Public Health, Faculty of Health and Medicine, University of Newcastle, Callaghan, Australia; 2 Conjoint Lecturer, Priority Research Centre for Health Behaviour, Faculty of Health and Medicine, University of Newcastle, Callaghan, Australia; 3 Affiliate, Hunter Medical Research Institute, New Lambton, Australia; 4 NHMRC Boosting Dementia Leadership Fellow, Health Behaviour Research Collaborative, School of Medicine and Public Health, Faculty of Health and Medicine, University of Newcastle, Callaghan, Australia; 5 NHMRC Boosting Dementia Leadership Fellow, Priority Research Centre for Health Behaviour, University of Newcastle, Callaghan, Australia; 6 NHMRC Boosting Dementia Leadership Fellow, Hunter Medical Research Institute, New Lambton, Australia; 7 Senior Research Officer, Health Behaviour Research Collaborative, School of Medicine and Public Health, Faculty of Health and Medicine, University of Newcastle, Callaghan, Australia; 8 Senior Research Officer, Priority Research Centre for Health Behaviour, University of Newcastle, Callaghan, Australia; 9 Senior Research Officer, Hunter Medical Research Institute, New Lambton, Australia; 10 PhD Candidate, Health Behaviour Research Collaborative, School of Medicine and Public Health, Faculty of Health and Medicine, University of Newcastle, Callaghan, Australia; 11 PhD Candidate, Priority Research Centre for Health Behaviour, University of Newcastle, Callaghan, Australia; 12 PhD Candidate, Hunter Medical Research Institute, New Lambton, Australia; 13 Senior Policy Analyst, Cancer Council New South Wales, Woolloomooloo, Australia

**Keywords:** Palliative care, general practice, qualitative research, health care delivery, primary health care

## Abstract

**Background:**

GPs can play a central role in palliative care delivery. However, little is known about their views on what constitutes best practice care at the end of life.

**Aim:**

To explore, in a sample of Australian GPs, their perceptions of best practice palliative care and their ideal role in its delivery.

**Design & setting:**

A qualitative interview study of 25 GPs practising in metropolitan and non-metropolitan locations in New South Wales, Australia.

**Method:**

Semi-structured telephone interviews were conducted. Data were analysed using qualitative content analysis.

**Results:**

Participants had a mean age of 51 years, and had practised between 3 and 38 years (mean 19 years). Best practice palliative care was perceived to be proactive and responsive to a wide range of patient and family needs. Many participants indicated a need for relational continuity, which involves GPs establishing a care pathway from diagnosis to palliation, coordinating care across the pathway, and collaborating with other healthcare providers. A number of participants perceived palliative care as a natural extension of primary care and indicated that best practice palliative care mainly requires experiential knowledge and good communication skills, rather than specialised medical knowledge. Participants listed a number of communication strategies to offer patients and their families choice and ongoing negotiation about the recommended treatments.

**Conclusion:**

This study provides novel in-depth insights into GPs’ perceptions of best practice palliative care. Future research should further investigate the identified features of care, and whether they can maximise the outcomes of patients and their families.

## How this fits in

Many patients do not receive best practice palliative care. GPs often play a key role in palliative care, and numerous studies have identified their views on barriers and enablers to best practice palliative care delivery. Yet, before recommendations can be made on how to overcome these barriers, it is necessary to understand GPs’ perceptions of what best practice palliative care is and their ideal role in its delivery. This qualitative study provides novel in-depth insights into GPs’ perceptions of best practice palliative care.

Best practice palliative care was described as being accessible, holistic, dynamic, integrated, and patient-centred. It involves coordination of care across the whole care pathway and with other healthcare providers. GPs perceived palliative care as a natural extension of primary care. These findings provide some guidance on how to establish palliative care pathways that involve multidisciplinary teamwork and ensure continuous and flexible care delivery which is tailored to the changing needs of individual patients and their families.

## Introduction

Best practice general practice has been described as patient-centred, continuing, comprehensive, and coordinated care to individuals, their families, and their communities.^1,2^ Caring for people nearing the end of their lives is considered a part of the core business of general practice.^3,4^ Palliative care can improve the quality of life of patients facing problems associated with life-limiting conditions through the prevention and relief of suffering. It can also reduce the use of futile treatment and caregiver burden. Thus, palliative care serves to ‘*maximise the quality of life and considers physical, social, financial, emotional, and spiritual distress’.* ^5^ While some GPs achieve this in their practice, others are finding it increasingly difficult to deliver palliative care.^6^ Structural changes such as the rise of ‘drop-in‘ medical practices may have, in part, contributed to a lack of clarity about GPs’ role in palliative care and variability in how GPs engage in palliative care.^6–8^ For example, a systematic review suggested that the roles of healthcare professionals, including GPs, in delivering palliative care are unclear to patients, carers, and professionals themselves.^9^

### Exploring GPs’ views on best practice palliative care can inform healthcare delivery

Several studies have sought GPs’ perceptions of barriers and enablers to the delivery of best practice palliative care.^10–14^ There have also been a number of policy papers highlighting the important features of delivering best practice palliative care.^5,15^ However, most GPs are still self-defining their role in palliative care delivery. Further research in this area is warranted to help provide recommendations on what is expected of GPs and how healthcare delivery could be improved).^16^ One way to achieve this is to better understand GPs’ perceptions of what constitutes best practice palliative care and how it can be delivered in clinical practice.

Despite this, most studies in this area have focused on the perspective of patients and families,^17,18^ or GPs delivering palliative care and their perceptions of their day-to-day tasks.^19–25^ Only one study has included specific questions on what GPs consider best practice palliative care,^26^ though this was not the primary aim of the study. It also did not involve Australian GPs, and findings may not generalise to an Australian setting due to differences in healthcare systems and social norms. The current study will help fill this gap. It provides data from Australian GPs working in metropolitan and non-metropolitan locations. Non-metropolitan GPs may play a greater role than urban GPs in the care of palliative patients, as non-metropolitan patients often have less access to specialist palliative care services.^27^ Seeking the views of GPs working in various geographical locations will provide in-depth insights into the range and interplay of factors associated with best practice palliative care.

### Aims

To explore qualitatively,in a sample of Australian GPs, their perceptions of best practice palliative care and GPs’ ideal role in its delivery.

## Method

### Design

A qualitative study was conducted involving 25 semi-structured phone interviews with qualified GPs practising in New South Wales, Australia. This was a sub-study of a larger research project which aimed to explore barriers and enablers to the delivery of high-quality palliative care in primary care settings in New South Wales, Australia. GPs’ views on barriers and enablers are reported in a separate article (Herrmann A *et al*, unpublished data, 2019). The present study focuses on GPs’ perceptions of the most important features of best practice palliative care, and the GP’s ideal role in providing this.

### Recruitment

A stratified random sample of 240 GPs (50% from metropolitan and 50% from non-metropolitan practices) was identified through the Australasian Medical Publishing Company database. It is the most comprehensive database of medical practitioners in Australia and includes information about approximately 76% of all practising Australian GPs. All 240 GPs were mailed an invitation containing an information sheet, interest form, and reply paid envelope. A follow-up telephone call was made by a research assistant 2 weeks after non-response. All consenting GPs were recruited to this study. GPs were reimbursed for their time with a $100 (AUD) gift card, mailed on completion of the interview.

### Data collection

Data were collected via semi-structured telephone interviews. Verbal consent to join the study and permission to record and transcribe each interview were requested prior to commencement (further information available from the authors on request). Interviews were conducted by two female researchers trained in qualitative research methods, with previous experience in interviewing and qualitative analysis (AH, AZ). The interviewer and participant did not know each other prior to the interview. An open stimulus was used, where participants were encouraged to express their views about best practice palliative care in the way they prefer, with as little interruption as possible from the interviewer. This initial narrative helped explore GPs’ views on the most important features of best practice care. Open-ended questions and probes were used to elicit topic areas not initially spoken about by participants.^28^ GPs were also asked for details about: age, sex, number of years practising, and the setting in which they provided palliative care, including the number of GPs and practice nurses in their practice (further information available from the authors on request). Wherever possible, standardised questions were used to seek information on GPs’ sociodemographic and work-related characteristics.

### Data analysis

Audio recordings of interviews were transcribed by a professional transcription service. An inductive qualitative content analysis approach was chosen to investigate GPs’ perceptions of best practice palliative care.^29^ This method was considered particularly suited to gain in-depth insights into participants’ perspectives which are grounded in the actual data, rather than researchers’ preconceived categories and theoretical perspectives.^28^ Each whole interview was considered as a unit of analysis. Researchers read the transcripts line by line and examined, compared, and categorised their content to apply a paraphrase or label (a ‘code‘) which described what was interpreted in each section as important. The codes were checked by another researcher. Based on the initial codes, more abstract categories were developed.^30^ The codes and categories were used to form a coding matrix that was reviewed by all members of the research team. Based on the coding matrix, threads of meaning were generated across categories (themes), and thus both latent and manifest content were analysed.^29^ The robustness of the conclusions was tested on the basis of each case by comparing codes within each interview, as well as independently of cases by comparing codes between interviews.^31^ Demographics are presented using summary statistics.

## Results

### Sample characteristics

Data were collected between November 2017 and February 2018. Of the initial sample of 240 GPs, 229 were eligible to participate. Eleven GPs were no longer practising at their recorded practice (*n* = 9) or no longer practising as a GP (*n* = 2). Twenty-five GPs consented to participate and completed an interview (11% response rate). Table 1 shows participant characteristics. GPs worked in practices with an average of seven GPs (range 1–13 GPs) and three nurses (range 0–8 nurses). Non-metropolitan GPs were more likely to participate (28% metropolitan versus 72% non-metropolitan; χ^2^ (3) = 5.4; *P* = 0.02). Five themes were identified, described in detail below.

**Table 1.** Participant characteristics

	**Mean (range**)	***n* (%)**
**Sex (*n* = 25**)		
Male		13 (52)
Female		12 (48)
**Age, years (*n* = 25**)		
30–39	51 (34–62)	3 (12)
40–49		7 (28)
50–59		11 (44)
60–69		4 (16)
**Practice location (*n* = 25**)		
Major city		7 (28)
Inner regional		13 (52)
Outer regional		4 (16)
Remote		1 (4)
**Years practising as GP (*n* = 2** **4** **)**		
<10	19 (3–38)	7 (29)
10–19		4 (17)
20–29		5 (21)
≥30		8 (33)
**Hours worked per week (*n* = 25**)		
<35	39 (21–60)	9 (36)
35–44		7 (28)
45–54		6 (24)
≥55		3 (12)
**Travel time to work, one way, minutes (*n* = 25**)		
<10	16 (3–75)	9 (36)
10–29		12 (48)
30–59		3 (12)
≥60		1 (4)
**Employment (*n* = 25**)		
Principal		10 (40)
Employee		5 (20)
Associate or contractor		10 (40)
**Language spoken when practising (*n* = 25**)		
English only		21 (84)
English and other		4 (16)
**Postgraduate education in palliative care (*n* = 25**)		
Yes		1 (4)
No		24 (96)
**Country of medical degree (*n* = 25**)		
Australia		19 (76)
Other		6 (24)
**GP fellowships (*n* = 25**)^a^		
Royal Australian College of GPs		19 (76)
Australian College of Rural and Remote Medicine		2 (8)
None		6 (24)

a Totals add to more than 100% as GPs may be fellows of more than one college (that is, associations that bring together medical practitioners of a particular medical subspecialty or geographical area).

### Best practice palliative care is patient-centred and accessible

GPs reported that best practice palliative care should be holistic and responsive to a wide range of patient and family needs. Many GPs indicated that care should not be *‘medicalised*‘ (Female, 7 years practising) but should focus on psychosocial support for the patient and their family. It was considered important to help patients and families engage in care delivery, for example, by enabling them to seek timely advice, anticipate healthcare issues, and use medical equipment. To help achieve this, GPs in the study said that palliative care should be accessible to patients and their families whenever and wherever they need it. Accessibility was considered important in terms of time of day and geographic location. For example, GPs reported the need for after-hours services staffed by GPs or experienced nurses. GPs acknowledged the importance of patients and families having access to palliative care services regardless of whether they lived in urban, regional, or rural communities:

*‘GPs tend to have a very holistic approach to patient management, where we do take into account their physical, their psychological, their social, and their family needs. I think that that extends to the end part of their life as well.’* (Female, 25 years practising)

*‘I think generally looking after the family as well as the patient and trying to support their carers. Trying to look at their mental wellbeing and their choices, as well as just at symptom control with medication and things like that.’* (Female, 14 years practising)

### Best practice palliative care as integrated, continuing part of the doctor–patient relationship

Best practice palliative care was perceived to be long-term and ongoing, rather than being restricted to the terminal phase. Many GPs emphasised the need for relational continuity that involves GPs establishing a care pathway from diagnosis to palliation, and coordinating care across the pathway. GPs reported that relational continuity would also apply to multidisciplinary teamwork, as building relationships and networking with other healthcare providers over a longer period of time may lead to more efficient care. This was considered important to ensure consistent health monitoring and treatment, and to create *‘a therapeutic relationship between a patient and one or more providers that spans various healthcare events and results in accumulated knowledge of the patient and care consistent with the patient's needs*‘ (Male, 32 years practising). GPs reported that relational continuity of care does not necessarily end with the death of the patient but may continue into post-mortem care, involving tasks such as removing the body and caring for the bereaved family.

### A proactive and dynamic approach is needed to deliver best practice palliative care

GPs stressed the importance of proactive care which anticipates and flexibly responds to the changing patient and family needs. To achieve this, GPs emphasised their role in facilitating discussions about end-of-life care early in patients’ care trajectory. Many GPs reported that patients should be given their prognosis before the very final stages of life to allow for planning of care. They indicated that comprehensive doctor–patient communication and tailored support early on in a patient’s care trajectory could facilitate the provision of best practice care later on. Regular healthcare assessments were considered to be an opportunity to commence discussions about advance care planning with older people who have multiple comorbidities but were not being provided with palliative care:

*‘I mean we do feel pressure with time as well, especially in the busy practice. But my philosophy of dealing with these sort of patients is that I spend a good half an hour, 40 minutes with them and I don't mind that. In fact, I feel for them so, therefore, I don't mind taking extra time to give them that early support. That can make a big difference to their ongoing treatment after that.’* (Male, 15 years practising)

A number of GPs recommended providing patients with a step-by-step plan for their care that could be renegotiated if needed. GPs indicated that it was important to continuously monitor and assess patients’ changing needs, and offer patients and their families’ choices about the recommended treatments. They thought this would give patients and their families a sense of control over their situation. GPs said that this may also help ensure that treatment decisions were made before the patient becomes acutely unwell, so they do not have to make important care decisions in a crisis situation. GPs considered themselves as facilitators of patients’ wishes, who would not only elicit patients’ preferences but also tailor care accordingly:

*‘So I give them a bit of a sequential sort of outline of the steps that it might be going to lead to. So they’re well aware that, okay, this is the journey that we’re going to go along.* […] *Then, of course, will also tell them what to be mindful about psychological symptoms that will arise. And, at any time that they don’t feel right, welcome to come in and have a chat, so that we can help it more.’* (Male, 15 years practising)

*‘You know whatever way they want to go I just let them know that they’re in the driver’s seat and I’m just there to facilitate it for them.’* (Male, 3 years practising)

### GPs as key players for the delivery of best practice palliative care

Many GPs described palliative care as a natural extension of primary care. They indicated that their profession has the clinical knowledge, skillset, and experience required to deliver palliative care. This was for a number of reasons, including GPs’ long-term relationship with many of their patients and their self-concept as a committed and caring listener and solver of people’s everyday problems. Many GPs said that this notion of a family doctor may differ from modern ‘corporate‘ GP practices that may lack continuity of care and emotional attachment. GPs also reported that the aim of palliative care was to comfort rather than cure, and make *‘that last part of their life as best as they possibly can with the best experience possible for the patient and the family*‘ (Female, 25 years practising). Some GPs said that palliative care could be a rewarding experience for the GP:

*‘My goal as a doctor is to make people’s life better or just relieve their suffering and help them. So in palliation, it's a really golden opportunity to do that.’* (Male, 3 years practising)

*‘It used to always puzzle me when I first went into general practice that people would come in and they’d tell you these terrible problems and you’d listen to them for 15 or 20 minutes and you wouldn’t have a clue what to do. Somehow, they’d leave happier than when they walked in because just they’d shared it and here was someone who took them seriously and was trying to help people.* […] *It’s the same with the palliative care thing. That listening to the patient and sharing their experience, that’s somehow very therapeutic.’* (Male, 30 years practising)

Palliative care was also perceived as something that is not *‘rocket science*‘ (Male, 35 years practising) and *‘that most doctors should be capable of doing well*‘ (Male, 30 years practising). It was seen as *‘old-fashioned*‘ (Male, 32 years practising), general rather than specialised care requiring a lot of experiential knowledge and good communication skills but relatively little technical expertise:

*‘I actually think GPs should run palliative care. I think it’s an area of medicine where they can be the specialists because it’s not just about putting in fancy lines and things. It's really about knowing the family towards being able to provide good care. I think it’s an area where GPs can be the specialists, can provide excellent care and should really.’* (Male, 35 years practising)

*‘I think it’s the art of medicine.* […] *It’s very clinical because even though I said I love tests, you’re trying not to do tests. You try not to do uncomfortable intrusive things and so it’s very clinical, which I guess is old-fashioned now. It involves the skills that we should all be good at with the communication and what have you. Whether that’s old-fashioned, it should be universal, I guess, shouldn’t it?’* (Male, 30 years practising)

*‘We all know the effect of malignancy, how they’re going to affect your body as a whole. So you may not need to explain to them specifically about that particular cancer. If you do have enough knowledge on that one, then, of course, you can explain more, but you don’t have to. But at least you should have enough knowledge in the whole palliation conditions.’* (Male, 15 years practising)

However, GPs also highlighted that some more complex cases require the input of palliative care specialists. GPs indicated that best practice palliative care should involve a multidisciplinary team of healthcare providers and services, including community services, allied health professionals, and palliative care physicians. Despite this, GPs reported that they had an important role to play in managing multidisciplinary teamwork and coordinating patient care. This was because GPs suggested that, compared to specialists, they were more accessible to patients and that their care usually involved less out-of-pocket costs and shorter waiting times. GPs said that they were often patients’ first point of contact at various stages of the care trajectory, which provided them with a better understanding of the variety and interplay of patients’ symptoms and needs, than a specialist may have:

*‘Certainly I do think we, as a GP, play a significant role in this* [=providing palliative care] *because we see them more often than they see the oncologist. Because whenever they feel anything change they always see us first and we are the first point of contact for them.’* (Male, 15 years practising)

*‘You're more accessible than the palliative care consultant specialist, both to the palliative care team as well as to the patient themselves. We can liaise with other allied health people if people need services such as physiotherapy or massage therapy or podiatry, all those things that might be required, we can organise those as well. So it’s sort of a general all-stop thing, we can easily offer access to the palliative care physician when we need advice. We’re relatively inexpensive.’* (Male, 30 years practising)

### Communication skills are essential to delivering best practice palliative care

GPs listed a number of communication strategies to facilitate palliative care delivery, including active listening, realistic communication about life expectancy and recommended care, focusing discussions on the benefits of palliation rather than giving patients false hopes for cure, and conveying that there are treatments available to keep them comfortable. GPs mentioned a need for consistency in how information is presented to patients to ensure they understand their diagnosis and treatments and can become involved in their healthcare decisions. GPs also indicated that it is important to ask patients about their wishes, rather than imposing their own beliefs on patients’ dying process. This would help ensure that palliative care is not reactive to family pressure at the expense of patients’ needs. GPs emphasised that home visits were no longer seen as a common service delivered by GPs, particularly in rural areas. Thus, it was considered important to educate patients and their families that they are not an inconvenience to GPs if they prefer to be visited or die at home:

*‘You've got to really listen to them, and also the family around them and what their wants are.’* (Female, 22 years practising)

*‘We’ve got an exercise physiologist who was able to really support the family to connect with her, and even though she had end-stage dementia she supported the family to move her gently as they were moving her, and I think that really supported her transition as well — her death, rather.* […] *It gave them a sense that they could actually be very active and support her that way and that they didn’t feel so powerless or so hopeless about that.’* (Female, 10 years practising)

## Discussion

### Summary

GPs in this sample identified key features that were considered most important to best practice palliative care. These included accessibility, a holistic, proactive, and dynamic approach to care, continuity of care, and excellent communication skills. While these features have been individually identified as important to palliative care,^32^ these findings add to the literature by explicitly outlining the role of these aspects, from the GP perspective, as interrelated and essential building blocks for achieving best practice patient-centred palliative care. The findings further raise novel aspects of GPs’ views on best practice palliative care. First, palliative care was seen as a natural extension of primary care which focuses on the whole care trajectory, from diagnosis to palliation. Also, GPs perceived themselves as palliative care experts, delivering anticipatory care coordination before patients become acutely unwell.

### Comparison with existing literature

Patient-centred care is driven by the ethos that care should be directed by patient needs and preferences.^33^ Therefore, proactive care which anticipates and plans for future care needs was considered key. Other studies have identified aspects of proactive care, such as anticipatory scripts and advance care planning, as important practices for ensuring patients’ needs are met at the end of life.^34,35^ GPs in the present study also deemed communication skills to be essential to delivering best practice care, particularly in ensuring that patients are informed about their prognosis and the choices they may face regarding their care. These views regarding the role of communication align with other studies which have asserted the key role of communication skills in eliciting patient needs and ensuring informed choice.^36^ For example, GPs indicated a need to discuss palliative care options early on in patients’ care trajectory. This is in line with previous studies suggesting that discussing treatment options with patients soon after diagnosis can make future discussions more succinct and thus lead to more efficient and effective care.^37^ Despite this, GPs in the current study acknowledged communication in palliative care to be difficult. Discussion around the transition from curative to palliative care was seen as particularly challenging, which aligns with previous studies in this area.^36^ Participants in the present study suggested that these difficult discussions may be facilitated by increasing awareness of the importance of tailored care planning among GPs and improving care processes. For example, patients could be provided with a written step-by-step plan for care delivery which can be renegotiated if needed. Also, patients could be given information about advance care planning at diagnosis of a life-limiting illness, or when conducting a routine health assessment of older patients. Specific barriers and enablers to best practice palliative care will be detailed in a separate article which is currently under review (Herrmann A *et al*, unpublished data, 2019).

These findings highlight GPs’ views that accessible care is a key component of best practice palliative care, which is not always achieved. For example, accessibility of after-hours care was seen to provide reassurance to patients and their families that their medical care and comfort needs are met in timely ways. However, GPs in this study indicated that accessibility of after-hours care is often lacking. While other studies have also identified the importance of accessibility of palliative care, this is often reported to be difficult to achieve in practice.^38–40^ For example, access to community palliative care is often poorer for people living in rural or remote areas,^41^ many GPs are unable to provide home visits,^42^ and better access to after-hours telephone and other services is needed.^43^ This suggests that accessibility should be improved if best practice palliative care is to be provided equitably, irrespective of patients’ geographic location. GPs in this study reported on what palliative care should look like in clinical practice. While the authors did not specifically ask, they infer that GPs in the study elaborated on *best practice* care, rather than *ideal* care (that is, what could be aspired to but not realistically achieved).

While acknowledging the important role of specialists and nurses and the need for multidisciplinary teamwork, responders in this study perceived that GPs can play a central but often underestimated role in the delivery of high quality palliative care. This view aligns with published literature that has advocated for a greater involvement of primary care in the delivery of palliative care.^8^ This is due to increasing concern of future insufficient specialist workforce capacity to meet the growing demands of palliative care.^44,45^ It is of interest to note, however, that GPs in the present study mainly reported patient-centred rather than economic or pragmatic reasons for emphasising the need for GPs to be key players in palliative care. GPs perceived that they were best placed to deliver palliative care given their knowledge of the patient and family, and ability to coordinate the different roles and responsibilities within multidisciplinary healthcare teams. GPs’ views of themselves as care coordinators align with studies that have reported GPs perceiving themselves as playing an important role in ensuring continuity and coordination of care for people with serious and complex illnesses.^46^ Therefore, GPs may be likely to support policy efforts to promote the role of GPs in palliative care service delivery.

### Strengths and limitations

This study helps fill a gap in the literature by providing important in-depth insights into GPs’ perceptions of best practice palliative care. Specifically, these findings raise two novel aspects of GPs’ views on best practice palliative care: palliative care as a natural extension of primary care along the care continuum; and GPs perceiving themselves as palliative care experts who ensure anticipating care coordination before patients become acutely unwell. This knowledge can help overcome well documented barriers to palliative care delivery. The findings of this study may provide some assistance for policy and practice in establishing palliative care pathways that involve multidisciplinary teamwork and ensure continuous and flexible care delivery that is tailored to the changing needs of individual patients and their families.

This study involved 25 GPs from metropolitan and non-metropolitan (regional and rural) areas in New South Wales, Australia. The inclusion of GPs from a broad geographic area was a strength of the study. However, the study findings may not be representative of all GPs, given the low consent rate and small sample size. It is likely that GPs who were already engaged in or interested in palliative care were more likely to participate. Thus, the sample may be biased by GPs who have experience and/or greater interest in palliative care. Also, the participation rate of GPs practising in non-metropolitan areas was higher than that of GPs practising in metropolitan areas. Further research should explore the views of GPs who do not engage in palliative care delivery and focus on implications of geographical locations on palliative care delivery.

Given that open-ended questions were used, there is a risk that some relevant issues were missed. However, this risk was decreased by asking participants a number of semi-structured questions and probes to elicit their views on different aspects of best practice care delivery. At the end of each interview, GPs were also asked whether they have any further comments or suggestions. This was to ensure that all relevant topic areas were covered, including those that GPs considered important but that had not been included in the interview guide. Specific guidance was not provided to GPs as to whether they should consider barriers to care when providing their views. Consequently, it cannot be ascertained whether or not, and to what extent, GPs considered such barriers when describing important features of best practice palliative care. Also, each piece of data was coded by one researcher and double-checked by another. Given that this approach has been used widely in the literature, it was considered a suitable and feasible method of data analysis. However, independent and parallel analysis with subsequent discussion could have helped further refine the emerging themes. While some authors have suggested that telephone interviews provide lower quality data than face-to-face interviews, a number of studies compared both modes of data collection and did not find any difference.^47–49^ In addition, telephone interviews were considered particularly suitable for this study as this made it easier for GPs to integrate the interviews into their busy schedules and reschedule the interviews if necessary. Conducting telephone interviews, rather than face-to-face interviews thus helped reduce research-related burden on participants.

### Implications for research and practice

The results of this study provide new insights into GPs’ views about specific features of best practice palliative care. Similar to other conceptualisations of palliative care,^50,51^ these findings suggest that GPs define best practice palliative care as being accessible, holistic, dynamic, integrated, and patient-centred. GPs perceived palliative care as a natural extension of primary care. The findings also indicate that there is a need for advocacy work to help educate clinicians, patients and the wider public about the role GPs can play in palliative care delivery. These results align with government end-of-life care strategies in Australia and worldwide, which aim to strengthen the role of GPs in palliative care delivery.^52,53^

This was an explorative study which provides some preliminary suggestions for how the identified features of care could be implemented in day-to-day practice in order to support the delivery of best practice palliative care. These suggestions include establishing palliative care pathways that involve multidisciplinary teamwork, and ensuring continuous and flexible care delivery which is tailored to the changing needs of individual patients and their families. The results may be used alongside literature detailing barriers and enablers to best practice palliative care provision, to develop and test strategies which could help maximise the outcomes of patients and their families. Such strategies would require further investigation in a larger sample before examining for effectiveness in clinical practice.
